# Correlations between horizontal jump and sprint acceleration and maximal speed performance: a systematic review and meta-analysis

**DOI:** 10.7717/peerj.14650

**Published:** 2023-02-01

**Authors:** Junlei Lin, Jie Shen, Junjie Zhang, Aiguo Zhou, Wenxia Guo

**Affiliations:** 1 School of Strength and Conditioning Training, Beijing Sport University, Beijing, China; 2 Capital University of Physical Education and Sports, Beijing, China; 3 Beijing University of Chemical Technology, Beijing, China

**Keywords:** Standing long jump, Horizontal drop jump, Triple jump, Bounding, Sprint

## Abstract

**Objectives:**

The purpose of this study is to determine the associations between horizontal jump and sprint acceleration, as well as maximal speed performance.

**Methods:**

A systematic literature search was performed using PubMed, MEDLINE (EBSCOhost), and Web of Science. The studies that were included in this review must meet the following criteria: (1) well-trained individuals over the age of 18 years old; (2) Pearson’s correlation coefficients between sprint time and horizontal jump distance were provided; (3) the sprint distance was limited to 0–100 m. The quality of the studies was assessed using a modified version of the Downs and Black Quality Index test. A random-effects model was used to determine the effect sizes, and heterogeneity between studies was examined using the Q statistic and I^2^.

**Results:**

From the identified 2,815 studies, 27 studies were included in this study (two from reference lists). The sprint time of the sprint acceleration phase was moderately and negatively correlated with the standing long jump (*r* =  − 0.45, *z* = 7.48, *p* < 0.001), single leg standing long jump (*r* =  − 0.48, *z* = 3.49, *p* < 0.001) and horizontal drop jump distance (*r* =  − 0.48, *z* = 3.49, *p* < 0.001), and was largely and negatively correlated with multiple jump distance (*r* =  − 0.69, *z* = 6.02, *p* < 0.001). Out of five studies assessed the standing triple jump, three studies reported significant positive association with the sprint acceleration performance. The sprint time of maximal speed phase was very largely and negatively associated with standing long jump distance (*r* =  − 0.73, *z* = 4.44, *p* < 0.001) and multiple jump distance (*r* =  − 0.76, *z* = 6.86, *p* < 0.001).

**Conclusions:**

This review indicates the moderate to very large associations between horizontal jump and sprint acceleration and maximal speed performance, and the highest magnitude of associations between them is found in the multiple jump. Moreover, compared to the sprint acceleration performance, there are greater associations between maximal speed performance and standing long jump and multiple jump distance.

## Introduction

Sprint capability is an essential element of athletic performance in a variety of sports, including soccer (Bangsbo, Mohr & Krustrup, 2006), rugby (Duthie et al., 2006), and tennis (Parsons & Jones, 1998). Based on the distance traveled, sprint ability can be classified into acceleration (0–30 m) and maximum speed (30–100 m) (Mero, Komi & Gregor, 1992). Professional rugby players are required to accelerate and sprint at their maximum speed in order to win games. Several sprints lasting approximately 3 s were performed during a single rugby match (Deutsch, Kearney & Rehrer, 2007). Furthermore, players need to complete numerous sprints, which required them to reach speeds of almost 90% of their maximum speed (Duthie et al., 2006). Athletes’ acceleration performance is more important than their maximal speed in other sports, such as basketball and soccer (Ben Abdelkrim, El Fazaa & El Ati, 2007; Cronin & Sleivert, 2005). Different physiological systems are involved in the development of sprint acceleration and maximum speed performance. The investigation of detailed variables will provide practitioners with some valuable information.

Sprint performance is determined by several factors including technique, spring-specific endurance, power and so on. A study conducted by Haugen, Breitschädel & Seiler (2019) found that maximal power output is strongly and negatively correlated with the 10 m and 40 m sprint time. Moreover, as the sprint speed increases, the demand for power output also increases. Jumping drill, such as vertical and horizontal jump, was a common method to assess and develop lower limb power. Jumping exercise enhances the neuronal and musculotendinous systems’ ability to create power through the stretch-shortening cycle (Markovic & Mikulic, 2010; Meylan & Malatesta, 2009). However, according to the training specificity hypothesis (Bachman, 1961) and the dynamic correspondence theory (Zweifel, 2017), the training drills chosen must have some features that are similar to those seen in specific sports. The closer the selected movements are to the specific sport, the more accurate the assessment will be and the more benefits the training will provide. The characteristics of specific sports include muscle action velocity, movement direction and muscle involved. It is notable that there exists some argument over the force-vector theory. The opponents considered that there are several “mechanical misconceptions” in this theory, and the most significant issue is that in complex movement like sprint, it primarily considered the direction of force relative to the global frame and not relative to the athlete, which is the most crucial factor (Fitzpatrick, Cimadoro & Cleather, 2019). Therefore, although vertical jumping reflects vertical power and horizontal jumping reflects horizontal power, it is hard to say which jumping will be greater correlated with sprint performance. However, there are significant differences in the contributions of the hip, knee, and ankle to horizontal and vertical jump performance. Hip and knee play a greater role in horizontal jump performance (44% and 43%, respectively) than in vertical jump performance (31% and 34%, respectively). This difference will influence the magnitude of associations between sprint performance and horizonal and vertical jump performance (Kotsifaki et al., 2021).

Standing long jump (Abbas, 2016; Banda et al., 2019), horizontal drop jump (Schuster & Jones, 2016), horizontal triple jump (Hudgins et al., 2013), and multi-step jump (steps >3) (Hennessy & Kilty, 2001) are all common horizontal jump drills. Notably, the abilities reflected in the various horizontal jump tasks varied. For example, the standing long jump requires explosive lower-body strength, whereas the multi-level jump, such as the sprint bound, highlights reactive strength. Thus, it is necessary to clarify the correlations between various horizontal jumping drills and sprint performance. Although the relationships between horizontal jump distance and sprint acceleration/maximal speed performance have been well examined in previous studies (Abbas, 2016), the findings have been demonstrated to be inconsistent. For example, in terms of sprint acceleration performance, the study conducted by Abbas (2016) found that there are significant negative correlations between standing long jump distance and 20 m sprint time in professional basketball athletes. Nevertheless, no significant correlations were observed between standing long jump and 10m and 3/4 court sprint (22 m) time in collegiate basketball players (Banda et al., 2019). Similarly, in terms of maximal speed performance, Hudgins et al. (2013) reported significant negative correlations between triple jump distance and 60 m and 100 m sprint time (*r* =  − 0.97 and −1.00, respectively, *p* < 0.05), while there were no significant relationships between 100 m sprint time standing long jump, standing triple jump, standing quintuple jump and 10-step jump distance (*r* = 0.33, 0.18, 0.25, 0.29, respectively, *p* > 0.05) in male sprinters (Kale et al., 2009). The disparity in outcome measures may be the explanation for these conflicting findings.

As a result, it is critical to synthesize existing evidence. Therefore, the purpose of this review was to determine the associations between the measures of horizontal jump and sprint acceleration/maximal speed performance in well-trained athletes.

## Material and Methods

The Preferred Reporting Items for Systematic Reviews and Meta-Analyses (PRISMA) statement was followed when conducting this meta-analysis (Moher et al., 2009). A review protocol was not pre-registered for this review.

### Search strategy

The electronic literature search was performed in PubMed, MEDLINE (EBSCOhost), and Web of Science up to January 9th, 2022. The following terms were searched in Boolean search syntax: (“jump” or “hop” or “reactive strength”) and (“sprint” or “speed”) and (“relationship study” or “association” or “correlation”). The research was limited to the English language and the human species. Moreover, the references list of the studies included in this review was searched for potentially relevant studies.

### Selection criteria

The following criteria had to be satisfied for studies to be considered for inclusion: (a) subjects who are over the age of 18; (b) well-trained subjects who are professional sports players or student athletes or healthy players with sports background; (c) the Pearson’s correlation coefficients between sprint time and horizontal jump distance were reported; (d) the sprint distance was limited to 0–100 m. Studies were excluded if: (a) they were non-peer-reviewed articles; (b) exact sprint time and jump distance were not reported; (c) only the abstract was provided. After eliminating the duplicated articles from the search results, two authors independently assessed the titles and abstracts of the leaving studies. The full articles were then reviewed based on the inclusion and exclusion criteria. If the first two reviewers (Z and S) couldn’t come to an agreement, a third reviewer (L) was approached.

### Data collection process

Each study included in this review was extracted for the following data on Microsoft Excel sheets: study (authors and publication year), subject characteristics (*e.g.*, number, age, gender, height, body weight, and athletic background), assessment tools (*e.g.*, 10 m, 20 m, standing long jump, horizontal drop jump, etc.) and main outcomes (correlation coefficients and *p*-value). Furthermore, horizontal jump tests of more than three steps were all classified as multiple jump tests.

### Quality assessment

Methodological quality was assessed using a modified version of the Downs and Black checklist (Bujalance-Moreno, Latorre-Román & García-Pinillos, 2019; Downs & Black, 1998; Fox et al., 2018) for assessing the methodological quality of randomized and nonrandomized healthcare interventions. Two independent authors examined studies included in this review based on the ten items of the assessment. The score of each item was using a Yes (1) or No (0), and a third rater was contacted to resolve contradictory results. The total score was 10, with higher scores indicating methodological quality.

### Statistical analyses

Associations between variables of horizontal jump and acceleration/maximal speed were analyzed in well-trained athletes using the Pearson product-moment correlation coefficient (*r*-value). Effect sizes were calculated following a three-stage process. Firstly, Pearson’s r values (r) were computed by Fisher’s z’ transformation: Z_r_ = 0.5 × ln($ \frac{1+\mathrm{r}}{1-\mathrm{r}} $), where ln is the natural logarithm (Cumming, 2013). Secondly, the sample size (n) of included studies was used to calculate the standard error (SE) according to the following formula: SE${}_{\mathrm{Z}}=\sqrt{ \frac{1}{n-3} }$. Finally, values of Z_r_ were back-transformed to Pearson’s r using the following formula: $r= \frac{e \left( 2\times Zr \right) -1}{e \left( 2\times Zr \right) +1} $, where e is the base of the natural logarithm (Cumming, 2013). Furthermore, when multiple effect sizes were reported for one variable in one study, an average Z_r_ data was used to back-transformed to Pearson’s *r*. Values of *r* ≤ 0.10 identified trivial, 0.11<*r* < 0.29 small, 0.30 <*r* < 0.49 moderate, 0.50 <*r* < 0.69 large, 0.70 <*r* < 0.89 very large (Hopkins et al., 2009).

Both the Q statistic and I^2^ were used to assess statistical heterogeneity. When an I^2^ value greater than 50% identified significant heterogeneity. Publication bias was assessed by funnel plots. Egger’s test was used to quantify bias when the number of studies within the analysis exceeded 10 (Sterne et al., 2011). A random-effects model was conducted using Stata software version 14.0 (Stata Corporation, College Station, TX, USA).

## Results

**Figure 1:**
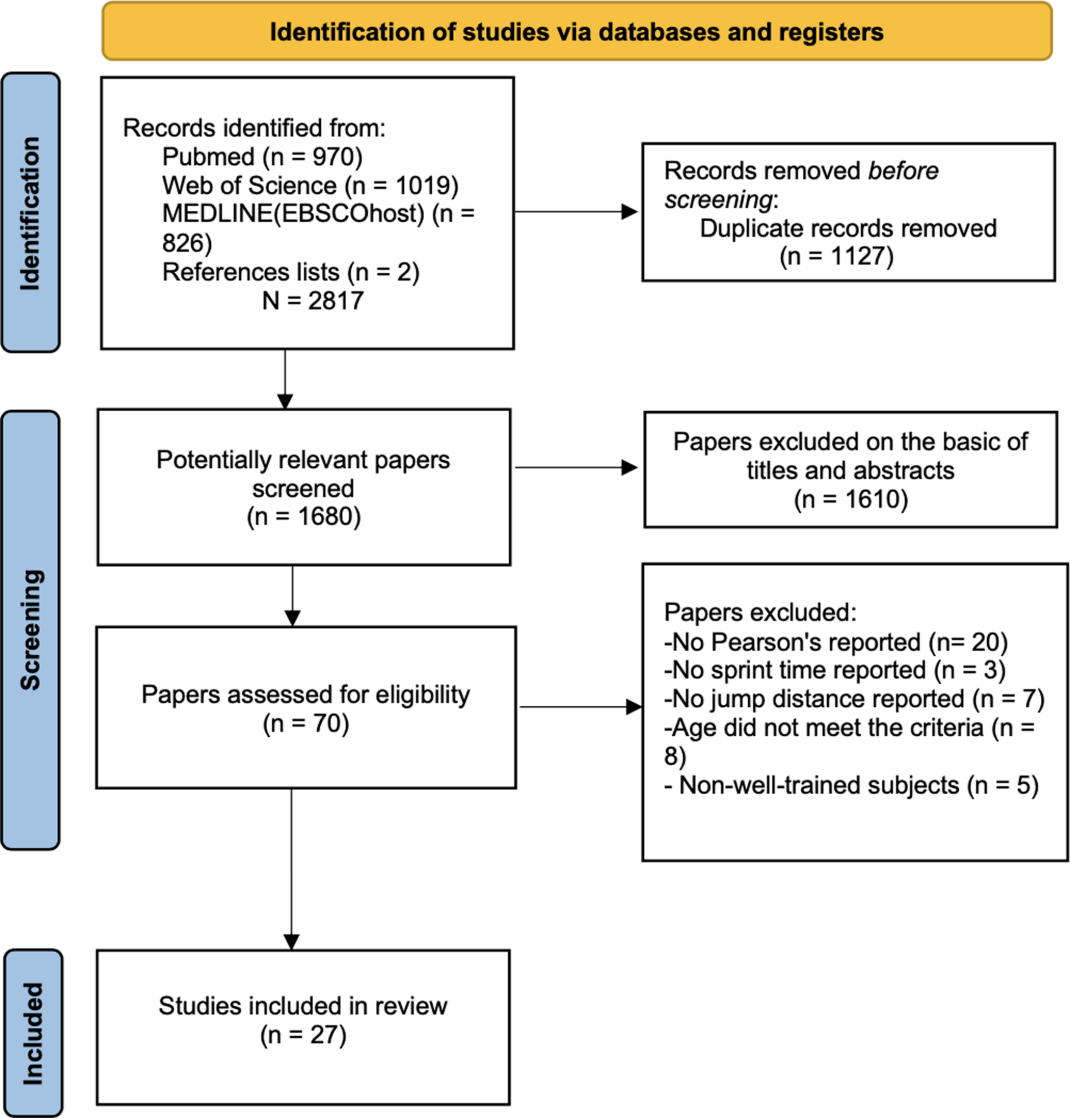
PRISM flow chart of the study selection process.

The initial search identified 2,817 studies (PubMed = 970; Web of Science = 1,019; MEDLINE (EBSCOhost) = 826; references list checks = 2). After removing 1,137 duplicates, the titles and abstracts of 1,680 studies were reviewed. The leaving 70 full texts were evaluated, and 42 studies remained. Ultimately, 27 studies were included in this review. The flow diagram illustrating the characteristics of the literature search is presented in Fig. 1.

### Methodological quality

The study’s quality assessment is shown in Table 1. The scores of the studies included in this review ranged from 6 to 9, with an average of 7.82. No trials were excluded because of the poor quality. All studies clearly described their purpose, primary outcomes, key findings, and mean ± SD in the data. Moreover, the statistical analysis was appropriate, and the main outcome measures were accurate.

**Table 1:** Methodological quality assessment of studies included in this study.

Number/Study	1	2	3	4	5	6	7	8	9	10	Score
(1) Abbas (2016)	1	1	1	1	1	1	0	1	1	1	9
(2) Banda et al. (2019)	1	1	1	1	1	1	0	1	1	1	9
(3) Chaouachi et al. (2009a)	1	1	1	1	1	1	0	1	1	1	9
(4) Habibi et al. (2010)	1	1	1	1	1	1	0	1	1	1	9
(5) Holm et al. (2008)	1	1	1	1	1	0	0	1	1	1	8
(6) Hudgins et al. (2013)	1	1	1	1	1	0	0	1	1	1	8
(7) Kale et al. (2009)	1	1	1	1	1	0	0	1	1	1	8
(8) Kulakowski et al. (2020)	1	1	1	1	1	1	0	1	1	1	9
(9) Lockie et al. (2014)	1	1	1	1	1	1	0	1	1	1	9
(10) Lockie, Dawes & Callaghan (2020)	1	1	1	1	1	1	0	1	1	1	9
(11) Loturco et al. (2015)	1	1	1	1	1	1	0	1	1	1	9
(12) Mackala, Fostiak & Kowalski (2015)	1	1	0	1	1	0	0	1	1	1	7
(13) Mackala et al. (2021)	1	1	1	1	1	0	0	1	1	1	8
(14) Maulder, Bradshaw & Keogh (2006)	1	1	0	1	1	1	0	1	1	1	8
(15) McCurdy et al. (2010)	1	1	0	1	1	0	0	1	1	1	7
(16) Popowczak et al. (2019)	1	1	1	1	1	0	0	1	1	1	8
(17) Robbins & Young (2012)	1	1	1	0	0	0	0	1	1	1	6
(18) Schuster & Jones (2016)	1	1	1	1	1	0	0	1	1	1	8
(19) Washif & Kok (2020)	1	1	1	1	1	1	0	1	1	1	9
(20) Yanci et al. (2014)	1	1	1	1	1	0	0	1	1	1	8
(21) Dietze-Hermosa et al. (2021)	1	1	1	1	1	0	0	1	1	1	8
(22) Hennessy & Kilty (2001)	1	1	0	1	1	0	0	1	1	1	7
(23) Chaouachi et al. (2009b)	1	1	1	1	1	0	0	1	1	1	8
(24) Almuzaini & Fleck (2008)	1	1	1	1	1	0	0	1	1	1	8
(25) Maulder & Cronin (2005)	1	0	1	1	1	0	0	1	1	1	7
(26) Meylan et al. (2009)	1	1	1	1	1	0	0	1	1	1	8
(27) Nesser et al. (1996)	1	1	1	1	1	0	0	1	1	1	8

**Notes.**

1. The objectives of the study were clearly reported, 2. The main outcomes to be assessed were clearly reported, 3. The characteristics of the participants were clearly reported, 4. The main findings were clearly reported, 5. The estimates of the random variability in the data for the main outcomes were clearly reported, 6. The actual probability values were clearly reported, 7. Can the participants represent the entire population, 8. If any of the results of the study were based on ‘data dredging,’ was this made clear?, 9. Were the statistical tests appropriate, 10. Were the main outcome measure accurate.

1 The item was clearly reported. 0 the item was not clearly reported

### Meta-analysis

Of the 28 studies included in this study, only five studies (Nos. 6, 7, 11, 23, 25) did not report acceleration performance and 10 studies (Nos. 6, 7, 11, 12, 16, 20, 22, 23, 25, 27) reported on maximal speed performance. Horizontal jump performance was reported as standing long jump (*N* = 17) (Nos. 1, 2, 8, 9, 10, 11, 12, 13, 15, 16, 17, 18, 21, 22, 24, 25, 26), horizontal drop jump (*N* = 4) (Nos. 5, 15, 19, 21), standing triple jump (*N* = 7) (Nos. 4, 6, 7, 13, 14, 21, 26) and multiple jump (*N* = 6) (Nos. 3, 12, 20, 23, 24, 27). The characteristics and number of the studies included in this review are shown in Table 2.

**Table 2:** The number and characteristics of studies included in this review.

Number/Study	Participants	Training status	Jump tests	Sprint tests	Main outcomes
No.1 Abbas (2016)	*N* = 16, Male	Professional basketball players	SLJ: cm 222.4 ± 18.3	20m: s 3.5 ± 0.2	SLJ VS 20m *r* = − 0.76, *p* = 0.001
						Age: yr 19.5 ± 0.8				
						Height: cm 180.2 ± 7.4				
	Weight: kg 72.1 ± 10.4				
No.2 Banda et al. (2019)	*N* = 12, Female	Collegiate basketball athletes	SLJ: m 1.99 ± 0.15	10m: s 1.93 ± 0.10 22m: s 3.59 ± 0.20	SLJ VS 10m *r* = − .0.289, *p* = 0.362 SLJ VS 22m *r* = − 0.478, *p* = 0.116
						Height: m 1.75 ± 0.09				
	Weight: kg 73.37 ± 17.30				
No.3 Chaouachi et al. (2009b)	*N* = 14, Male	Healthy basketball players	5 Jump: m 13.0 ± 0.6	5m: s 0.82 ± 0.05 10m: s 1.70 ± 0.06 30m: s 4.16 ± 0.11	5 Jump VS 5m: *r* = − 0.41, *p* = 0.11
					Height: cm 195.6 ± 8.3				VS 10m *r* = − 0.65, *p* = 0.02*
	Weight: kg 94.2 ± 10.2				VS 30m *r* = − 0.74, *p* = 0.01*
No. 4 Habibi et al. (2010)	*N* = 15, Male Age: yr 21.89 ± 3.26, Height: m 1.72 ± 3.20, Weight: kg 61.35 ± 11.40	Iranian track sprinters	SL-SLJ Front leg: m 1.97 ± 0.02	10m: s 1.90 ± .07	SL-SLJ VS 10m Font leg: *r* = − 0.74, *p* = 0.021
							Back leg: m 1.93 ± 0.18		Back leg: *r* = − 0.76, *p* = 0.017
							SL-STJ Front leg: m 6.63 ± .57		SL- STJ VS 10m Front leg: *r* = − 0.84, *p* = 0.004
			Back leg: m 6.50 ± .57		Back leg: *r* = − 0.89, *p* = 0.001
No. 5 Holm et al. (2008)	*N* = 20, Male Age: yr 22 ± 3 Height: cm 180 ± 7 Weight: kg 80 ± 9	Team sports players (primarily touch football, rugby, and basketball)	SL-HDJ (20 cm box): cm 171 ± 15	5m: s 1.13 ± 0.05 10m: s 1.87 ± 0.07 25m: s 3.78 ± 0.15 5–10m: s 0.74 ± 0.03 10–25m: s 1.90 ± 0.08	SL-HDJ VS 5m *r* = − 0.55, *p* < 0.01 SL-HDJ VS 10m *r* = − 0, 61, *p* < 0.01 SL-HDJ VS 25m *r* = − 0.51, *p* < 0.01 SL-HDJ VS 5–10m *r* = − 0.54, *p* < 0.01 SL-HDJ VS 10–25m *r* = − 0.40, *p* < 0.05
NO. 6 Hudgins et al. (2013)	N = 10, 5 male, 5 female Height: m 1.72 ± 10.26 Weight: kg 67.80 ± 10.83	Sprinters	STJ: m 8.24 ± 1.32	60m: ( *n* = 8) 7.28 ± 0.78 100m: ( *n* = 6) 11.25 ± 0.87	STJ VS 60m *r* = − 0.97, *p* < 0.05 STJ VS 100m *r* = − 1.0, *p* < 0.05
NO. 7 Kale et al. (2009)	N = 21, Male Age: yr 20.4 ± 1.9 Height: cm 175.8 ± 5.3 Weight: kg 70.3 ± 5.8	Sprinter	STJ: m 7.89 ± 0.56	100m: s 11.62 ± 0.41	STJ VS 100m *r* = 0.18, *p* > 0.05
NO. 8 Kulakowski et al. (2020)	N = 17, Female Age: yr 18 ± 0.7 Height: cm 162.4 ± 4.8 Weight: kg 62.5 ± 8.8	Division II collegiate lacrosse players	SLJ: cm 59.4 ± 6.0	10m: s 1.9 ± 0.1 30m: s 5.0 ± 0.3	SLJ VS 10m *r* = − 0.471, *p* = 0.06 SLJ VS 30m *r* = − 0.528, *p* = 0.03
NO. 9 Lockie et al. (2014)	N = 30, Male Age: yr 22.60 ± 3.86; Height: m 1.80 ± 0.07; Weight: kg 79.03 ± 12.26	Recreational team-sport athletes	SLJ: m left leg: 2.05 ± 0.19 right leg: 2.03 ± 0.17	5m: s 1.033 ± 0.075 10m:s 1.760 ± 0.010 20m:s 3.039 ± 0.164s	SLJ-Left VS 5m *r* = − 0.56, *p* = 0.001 VS 10m *r* = − 0.66, *p* < 0.001 VS 20m *r* = − 0.73, *p* < 0.001 SLJ-Right: VS 5m *r* = − 0.46, *p* = 0.01 VS 10m *r* = − 0.57, *p* = 0.001 VS 20m *r* = − 0.65, *p* < 0.001
NO. 10 Lockie, Dawes & Callaghan (2020)	N = 15, Female Height: cm 178.13 ± 8.96 Weight: kg 70.18 ± 7.58	Collegiate volleyball players	SLJ: cm 203.71 ± 26.03	10m: s 2.03 ± 0.12 20m: s 3.51 ± 0.16	SLJ VS 10m *r* = − 0.21, *p* = 0.444 SLJ VS 20m *r* = − 0.21, *p* = 0.454
NO. 11 Loturco et al. (2015)	N = 14, Male Age: yr 24.9 ± 3.8 Height: cm 178.7 ± 6.4 Weight: kg 77.8 ± 8.5	Elite sprinters	SLJ: m 2.90 ± 0.11	100m: s 10.49 ± 0.19	SLJ VS 100m *r* = − 0.81, *p* < 0.01
NO. 12 Mackala, Fostiak & Kowalski (2015)	N=11, Male Age:21.7 ± 1.08 yrs; Height:180.8 ± 6.98 cm; Weight:76.6 ± 7.62 kg	High performance sprinters	SLJ: cm 285.71 ± 15.94 5 jumps: m 14.65 ± 1.01 10 jump: cm 30.68 ± 1.66	10m: s 1.89 ± 0.08 30m: s 3.93 ± 0.24 100m: s 11.14 ± 0.36	SLJ VS 10m *r* = − 0.74 VS 30m *r* = − 0.70 VS 100m *r* = − 0.82 5 jumps VS 10m *r* = − 0.65 VS 30m *r* = − 0.62 VS 100m *r* = − 0.81 10 jumps VS 10m *r* = − 0.71 VS 30m *r* = − 0.67 VS 100m *r* = − 0.83
NO. 13 Mackala et al. (2021)	N = 66, Female: *n* = 22 Age: yr 20.18 ± 1.27 Weight: kg 62.23 ± 7.02 Height: cm 166.78 ± 5.29 Male: *n* = 44 Age: yr21.26 ± 1.78 Weight: kg 78.49 ± 7.94, Height: cm 182.18 ± 6.32	Healthy players	SLJ: m 2.62 ± 0.18 (male) 2.15 ± 0.09 (female) STJ: m 7.41 ± 0.55 (male) 6.35 ± 0.41 (female)	Male: s 10m: 1.84 ± 0.09 20m: 3.11 ± 0.13 30m: 4.30 ± 0.17 Female: s 10m: 2.01 ± 0.09 20m: 3.44 ± 0.14 30m: 4.82 ± 0.17	SLJ VS 10m male: *r* = − 0.181, female: *r* = − 0.510^*^ STJ VS 10m male: *r* = − 0.416^*^ female: *r* = − 0.253. SLJ VS 20m male: r = -0.557**, female: *r* = − 0.559 STJ VS 20m male: *r* = 0.476^**^ female: *r* = 0.606^**^ SLJ VS 30m male: *r* = − 0.461**, female: *r* = − 0.559^**^ STJ VS 30m male: *r* = − 0562.^**^ female: *r* = − 0.453**
NO. 14 Maulder, Bradshaw & Keogh (2006)	N = 10, Male Age: yr 20 ± 3 Height: m 1.82 ± 0.06 Weight: kg 76.7 ± 7.9	Track sprinters	SL-SLJ: m Front: 2.09 ± 0.09 Back: 2.10 ± 0.10 SL-STJ: m Front: 6.90 ± 0.21 Back: 6.90 ± 0.40	10m: s 2.04 ± 0.06	10m VS SL-SLH Front: *r* = − 0.30, *p* = 0.435 VS back *r* = − 0.23, *p* = 0.548 VS SL-STJ Front: *r* = 0.24, *p* = 0.532 Back: *r* = − 0.33, *p* = − 0.392
NO. 15 McCurdy et al. (2010)	N = 15 Age: yr 20.19 ± 0.91 Weight: kg 61.65 ± 7.7	National Collegiate Athletic Association (NCAA)	SLJ: m 1.47 ± 0.11 SL-SLJ: m 1.33 ± 0.11 HDJ (40 cm): m 1.42 ± 0.15 SL-HDJ (20 cm): m 1.39 ± 0.15	10m: s 2.31 ± 0.25 25m: s 4.52 ± 0.20	Left: SL-SLJ VS 10m: *r* = − 0.11 VS 25m: *r* = 0.15 SL-HDJ VS 10m: *r* = − 0.40 VS 25m: *r* = 0.15 Right: SL-SLJ VS 10m: *r* = − 0.22 VS 25m: *r* = − 0.39 SL-HDJ VS 10m: *r* = − 0.50 VS 25m: *r* = − 0.13 Pooled: SL-SLJ VS 10m: *r* = − 0.18 VS 25m: *r* = − 0.12 SL-HDJ VS 10m: *r* = − 0.40 VS 25m: *r* = 0.02
NO. 16 Meylan et al. (2009)	N = 80, Male, *N* = 44; Age: yr 20.9 ± 4.5 Weight: kg 78.1 ± 10.5, Height: cm 180.1 ± 7.0 Female: *N* = 36; Age: yr 19.7 ± 2.0 Weight: kg 62.1 ± 7.8 Height: cm 166.9 ± 6.0	Physical education university students	SLJ: m Male: 168.9 ± 19.3 Female: 134.6 ± 12.79	10m: s Male: 1.85 ± 0.08 Female: 2.11 ± 0.10	Male: *r* = − 0.65^**^ Female: *r* = − 0.339*
NO. 17 Popowczak et al. (2019)	N = 60, Male Age: yr 17.4 ± 0.7 Height: cm 176.3 ± 6.1 Weight: kg 68.1 ± 8.9	Soccer players	SLJ: cm 230.45 ± 13.70	10m: s 2.495 ± 0.104 30m: s 5.019 ± 0.179	SLJ VS 10m *r* = − 0.21 SLJ VS 30m *r* = − 0.24
NO. 18 Robbins & Young (2012)	N = 1136, Weight:kg 92.0 ± 6.01 to 136.8 ± 10.44 kg	National Collegiate Athletic Association Division I teams	SLJ	36.6m 18.3m 9.1m Flying 18.3m	SLJ VS 36.6m, 18.3m, 9.1m, Flying 18.3m All positions: *r* = − 0.467, *r* = − 0.428, *r* = − 0.353, *r* = − 0.353 lineman: *r* = − 0.394, *r* = − 0.332, *r* = − 0.324, *r* = − 0.327 Tightened: *r* = − 0.426, *r* = − 0.419, *r* = − 0.323, *r* = − 0.237 Linebacker *r* = − 0.578, *r* = − 0.550, *r* = − 0.394, *r* = − 0.415 Running back *r* = − 0.491, *r* = − 0.555, *r* = − 0.46*p*, *r* = − 0.2000 Quarterback *r* = − 0.425, *r* = − 0.395, *r* = − 0.213, *r* = − 0.284 Wide receiver *r* = − 0.257, *r* = − 0.193*r* = − 0.050, *r* = − 0.194
NO. 19 Schuster & Jones (2016)	N = 19, Male Age: yr 22.5 ± 3.2, Height: cm 181.1 ± 6.7, Weight: kg 80.3 ± 9.6	Collegiate team sport (Soccer and Rugby) athletes	HDJ (20 cm box): m 1.72 ± 0.33	0–5m: s 1.02 ± 0.04s 0–10m: s 1.74 ± 0.63 0–15m: s 2.44 ± 0.06 0–20m: s 3.09 ± 0.07 5–10m: s 0.74 ± 0.01 10–15m: s 0.74 ± 0.01 15–20m: s 0.66 ± 0.01	HDJ VS 0–5m *r* = − 0.66^**^ VS 0–10m *r* = − 0.57^**^ VS 0–15m *r* = − 0.66^**^ VS 0–20m *r* = 0.66^**^ VS 5–10m *r* = − 0.63^**^ VS 10–15m *r* = − 0.62^**^ VS 15–20m *r* = − 0.66**
NO. 20 Washif & Kok (2020)	N = 11, Male Age: yr 17.8 ± 1.3 Height: m 1.72 ± 0.06 Weight: kg 66.05 ± 6.10	Track and field sprinter	Unilateral horizontal 10 jumps: m 22.30 ± 1.43	10m: s 1.72 ± 0.04 30m: s 3.90 ± 0.09 50m: s 5.93 ± 0.15 10–30m:s 2.18 ± 0.06	10 jump VS 10m *r* = − 0.28, *p* = 0.402 VS 30m *r* = − 0.53, *p* = 0.093 VS 50m *r* = − 0.59, *p* = 0.059 VS 30m *r* = − 0.60, *p* = 0.054
NO. 21 Yanci et al. (2014)	N=39, Male Age: yr 22.9 ± 2.8, Height: cm 179.9 ± 6.01 Weight: kg 77.0 ± 8.3	Soccer players	SLJ: m 1.99 ± 0.15 D-SLJ: m 1.8 0 ± 0.13 ND-SLJ: m 1.81 ± 0.12 AS-SLJ: m 2.39 ± 0.14 D-HDJ: m 1.77 ± 0.12 ND-HDJ: m 1.85 ± 0.14 D-STJ: m 6.69 ± 0.39 ND-STJ: m 6.82 ± 0.43	5m: s 0.99 ± 0.03 10m:s 1.70 ± 0.05 20m:s 2.34 ± 0.06	SLJ, D-SLJ, ND-SLJ, AJ-SLJ, D-HDJ, ND-HDJ, D-STJ, ND-STJ. VS 5m *r* = − .047**, *r* = − 0.16, *r* = − 0.45*, *r* = − 0.82, *r* = − 0.30, *r* = − 0.26, *r* = − 0.86, *r* = − 0.29 VS 10m *r* = − 0.44**, *r* = − 0.13, *r* = − 0.41*, *r* = − 0.25, *r* = − 0.36*, *r* = − 0.35, *r* = − 0.27, *r* = − 0.41^*^ VS 15m *r* = 0.50**, *r* = − 0.22, *r* = − 0.47**, *r* = − 0.32, *r* = − 0.42*, *r* = − 0.39*, *r* = − 0.36, *r* = − 0.52**
NO. 22 Dietze-Hermosa et al. (2021)	N = 25 Height: cm 172 ± 9 Weight: kg 69.88 ± 9.77	Division I track and field athletes	SLJ: m 2.58 ± 0.32	30–35m: s 0.57 ± 0.04 30-40m: s 1.13 ± 0.08 30–45m: s 1.69 ± 0.12 30–50m: s 2.25 ± 0.16 30–55m: s 2.80 ± 0.20 30–60m: s 3.36 ± 0.26	SLJ VS 30–35m *r* = − 0.838^**^ VS 30–40m *r* = − 0.830^**^ VS 30–45m *r* = − 0.822^**^ VS 30–50m *r* = − 0.798^**^ VS 30–55m *r* = − 0.809^**^ VS 30–60m *r* = − 0.807^**^
NO. 23 Hennessy & Kilty (2001)	N = 17, Female Age: yr 17.6 ± 2.2 Height: cm 167.7 ± 3.7 Weight: kg 59.9 ± 7.2	Nationally ranked in sprint and hurdle events	5 Jump: m 10.98 ± 0.76	30m: s 4.58 ± 0.17 100m: s 12.90 ± 0.61	5 Jump VS 30m *r* = − 0.79^*^ VS 100m *r* = 0.49
NO. 24 Chaouachi et al. (2009a)	N = 21, Age: yr 24.3 ± 3.4, Weight: kg 88.6 ± 7.5 Height: m 1.89 ± 5.5	The senior Tunisian national handball team	SLJ: m 2.49 ± 0.16 D-SLJ: m 2.33 ± 0.16 ND-SLJ:m 2.21 ± 0.18	5m: s 1.17 ± 0.05 10m:s 1.93 ± 0.07 20m:s 4.44 ± 0.14	SLJ VS 5m, 10m, 30m *r* = − 0.38, *p* = 0.04, *r* = − 0.39, *p* < 0.04, *r* = − 0.45, *p* < 0.02 D-SLJ VS 5m, 10m, 30m: *r* = − 0.73, *p* < 0.001^**^ r = − 0.61, *p* < 0.001^*^ r = − 0.80, *p* < 0.001 ND-SLJ VS 5m, 10m, 30m: *r* = − 0.58, *p* < 0.001^**^ r = − 0.51, *p* < 0.01^*^ r = − 0.65, *p* < 0.001**
NO. 25 Almuzaini & Fleck (2008)	N = 38 Age: yr 21.66 ± 1.66, Height: cm 169.89 ± 6.34 Weight: kg 62.92 ± 8.68	Physical education students	SLJ: cm 213.70 ± 19.19	50m: s 7.12 ± 0.41 100m: s 13.54 ± 0.83	SLJ VS 50m *r* = − 0.45^*^ SLJ VS 100m *r* = − 0.422^*^
NO. 26 Maulder & Cronin (2005)	N = 18, Male Age: yr 25.1 ± 4.3 Weight: kg 78.8 ± 9.3 Height: cm 176.8 ± 5.1	Subjects involved in several sports	Horizontal squat jump: cm D: 1.596 ± 0.139 ND: 1.617 ± 0.136 SLJ: D: 1.642 ± 0.147 ND: 1.624 ± 0.177 STJ: m D: 5.105 ± 0.740 ND: 5.116 ± 0.657	20m	20m VS Horizontal squat jump *r* = − 0.73, *p* = 0.001 VS SLJ *r* = − 0.74, *p* < 0.001 VS STJ *r* = − 0.86, *P* < 0.001
NO. 27 Nesser et al. (1996)	N=20, Male Age: yr 23.4 ± 2.2 Weight: kg 79.9 ± 10.1 Height: cm 173.9 ± 5.44	University students and staff.	5 Jump: m 11.7 ± 1.1	40m: s 5.87 ± 0.32	5 Jump VS 40m *r* = − 0.81*

**Notes.**

N number yr years SLJ standing long jump D dominant ND non-dominant As with arm swing HDJ horizontal drop jump STJ standing triple jump yr year kg kilogram m meter cm centimeter SL single leg VS correlate with

* *p* < 0.05

** *p* < 0.001.

### Associations with standing long jump

**Figure 2:**
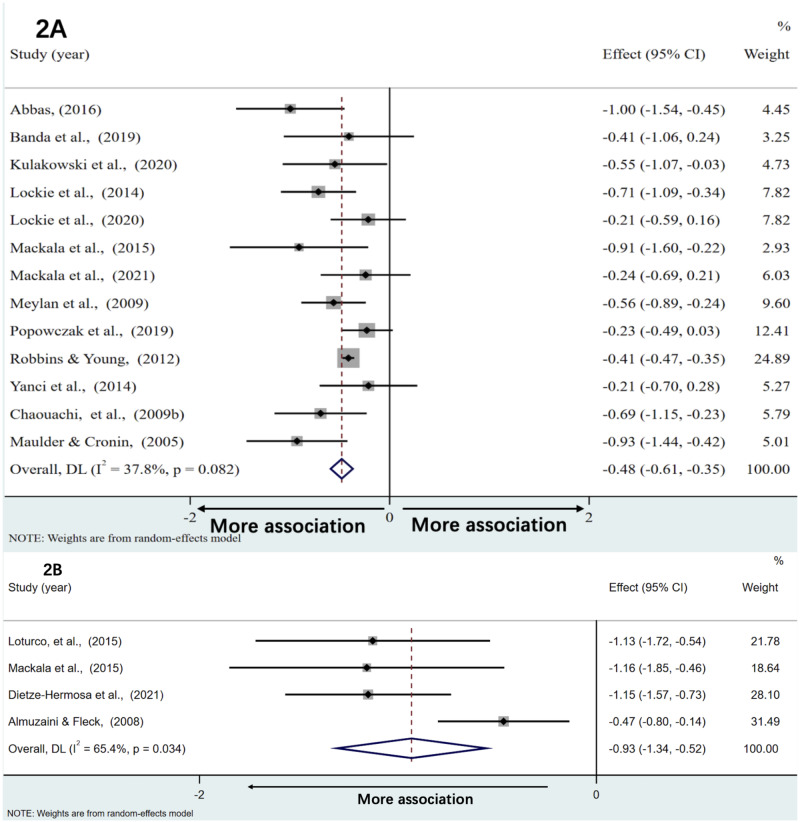
The associations between standing long jump and the sprint time of acceleration phase and maximal speed phase, respectively. The associations of standing long jump with acceleration (A) and maximal speed performance (B).

Figures 2A and 2B show forest plots of correlations between standing long jump and acceleration and maximal speed performance, respectively. Weighted effect values amounted to −0.48 [95% CI: −0.61,−0.35] (*Q* = 19.30; *p* = 0.08, *I*^2^ = 37.8%) for acceleration and −0.93 [95% CI: −1.34, −0.52] (*Q* = 8.68; *P* = 0.034, *I*^2^ = 65.4%) for results of maximal speed. Back-transformed r values of −0.45 [95% CI:−0.54,−0.34] (*Z* = 7.48; *p* < 0.001) and −0.73 [95% CI:−0.87,−0.48] (*Z* = 4.44; *p* < 0.001) demonstrated moderate and very large negative correlations with sprint time of the acceleration phase and the maximal speed phase, respectively.

### Associations with horizontal drop jump

**Figure 3:**
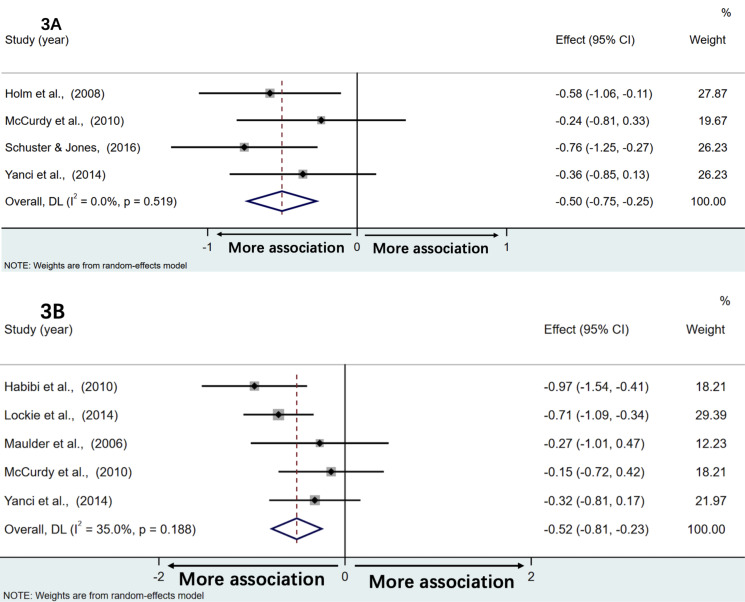
The associations between the sprint time of acceleration phase and horizontal drop jump and single leg standing long jump distance, respectively. The association of acceleration performance with horizontal drop jump distance (A) and single leg standing long jump distance (B).

Figure 3A illustrates a forest plot of the correlations between horizontal drop jump and the sprint time of acceleration phase. Weighted effect value was −0.50 [95% CI:−0.75,−0.25] (*Q* = 2.27; *P* = 0.52, *I*^2^ = 0.0%). Back-transformed r values of −0.46 [95% CI:−0.64,−0.25] (*Z* = 3.93; *P* < 0.001) demonstrated moderate and negative correlations with the sprint time of acceleration phase. However, no studies reported correlations between horizontal drop jump and maximal speed performance.

### Associations with single leg standing long jump

Figure 3B illustrates a forest plot of associations of single leg standing long jump distance with the sprint time of acceleration phase. Meta-analysis showed that weighted effect values amounted to −0.52 [95% CI:−0.81,−0.23] (*Q* = 6.15; *P* = 0.19, *I*^2^ = 35.0%). Back-transformed r values of −0.48 [95% CI:−0.67, −0.22] (*Z* = 3.49; *P* < 0.001) demonstrated moderate and negative correlations with the sprint time of the acceleration phase. Again, no studies explored correlations between single leg standing long jump and maximal speed performance.

### Associations with the standing triple jump

Given the high degree of heterogeneity, we had to conduct a qualitative analysis of the associations between the standing triple jump and sprint performance. Seven studies examined the associations between the standing triple jump distance and sprint time (Habibi et al., 2010; Hudgins et al., 2013; Kale et al., 2009; Mackala et al., 2021; Maulder & Cronin, 2005; Maulder, Bradshaw & Keogh, 2006; Yanci et al., 2014).

Five studies evaluated acceleration performance (Habibi et al., 2010; Mackala et al., 2021; Maulder & Cronin, 2005; Maulder, Bradshaw & Keogh, 2006; Yanci et al., 2014), while two assessed maximal speed performance (Hudgins et al., 2013; Kale et al., 2009). In terms of acceleration, 5 m (Yanci et al., 2014), 10 m (Habibi et al., 2010; Mackala et al., 2021; Maulder, Bradshaw & Keogh, 2006; Yanci et al., 2014), 15 m (Yanci et al., 2014), and 20 m (Mackala et al., 2021; Maulder & Cronin, 2005), and 30 m (Mackala et al., 2021) sprint time were evaluated in these studies. Notably, subjects included in these studies were sprinters and players from different sports backgrounds. Therefore, there were small variations in the sample. Significant and very large negative correlations between the standing triple jump distance and 10 m sprint time were observed in elite sprinters (Habibi et al., 2010) and in healthy males with different sports backgrounds (Mackala et al., 2021). In contrast, no significant correlations were reported in track sprinters (Maulder, Bradshaw & Keogh, 2006). Moreover, Yanci et al. (2014) examined associations between dominant and non-dominant side standing triple jump distance and 5 m, 10 m, and 15 m time in soccer players. Significant negative correlations were only observed between non-dominant side standing triple jump distance and 10 m and 20 m time (Yanci et al., 2014). Furthermore, significant and very large negative associations between the standing triple jump and 20 m time were observed in one study (Maulder & Cronin, 2005), which included subjects in several sports.

Two studies evaluated associations between maximal speed and the standing triple jump performance in sprinters. However, there were conflicting results. Hudgins et al. (2013) explored relationships between the standing triple jump distance and 60 m and 100 m time. Significant and very large negative correlations were observed (Hudgins et al., 2013). In contrast, no significant associations with 100 m time were reported in another study (Kale et al., 2009).

### Associations with multiple jump

**Figure 4:**
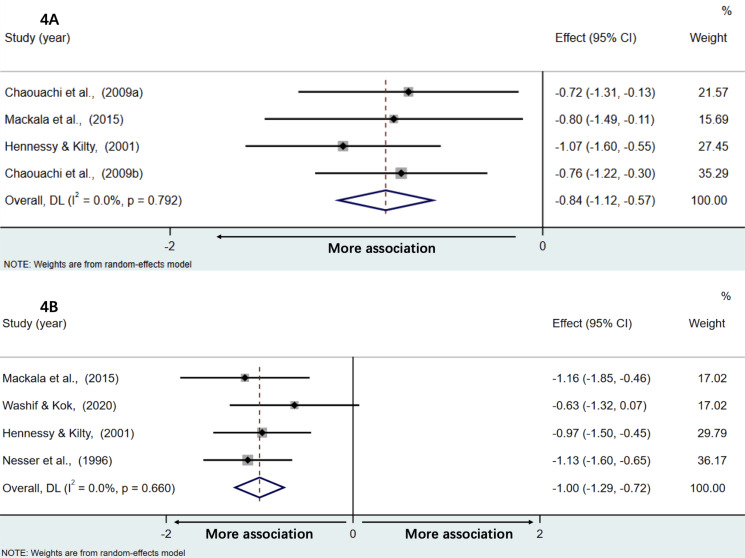
The associations between multiple jump distance and the sprint time of acceleration phase and maximal speed phase, respectively. The association of multiple jump distance with acceleration performance (A) and maximal speed performance (B).

Figures 4A and 4B present forest plots of associations of multiple jump distance with the sprint time of acceleration phase and maximal speed phase, respectively. Weighted effect values amounted to −0.84 [95% CI:−1.12,−0.57] (*Z* = 6.02; *P* < 0.001, *Q* = 1.04; *P* = 0.79, *I*^2^ = 0.0%) for acceleration and −1.00 [95% CI:−1.29,−0.72] (*Z* = 6.86; *p* < 0.001, *Q* = 1.60; *P* = 0.66, *I*^2^ = 0.0%) for results of maximal speed. Both back-transformed r values of −0.69 [95% CI:−0.81,−0.51] and −0.76 [95% CI:−0.86,−0.62] demonstrated large and very large negative correlations with the sprint time of acceleration phase and the maximal speed phase, respectively.

### Bias

First, we assessed bias in studies using funnel plots. The visual check confirmed that all plots were symmetrical, indicating that publication bias may not be evident. All funnel plots were presented in Figs. S5–S10. Furthermore, Egger’s test was used to determine the magnitude of publication bias in the analysis, which included more than ten studies. Egger’s test suggested there was no significant bias for the associations of standing long jump with acceleration performance (*t* =  − 1.61, *p* = 0.14).

## Discussion

This meta-analysis systematically synthesized and quantified previous studies on the correlations between horizontal jump distance and sprint acceleration and maximal speed performance. The results of this study demonstrated the sprint time of the acceleration phase is moderately and negatively correlated with standing long jump, single leg standing long jump, and horizontal drop jump distance, and largely and negatively correlated with multiple jump distance. The sprint time of the maximal speed phase is very largely and negatively correlated with standing long jump and multiple jump distance. Moreover, out of five studies assessed the standing triple jump, three studies reported significant positive association with the sprint acceleration performance. Therefore, this review indicates the moderate to very large associations between horizontal jump and sprint acceleration and maximal speed performance, and the highest magnitude of associations between them is found in the multiple jump. Moreover, compared to the sprint acceleration performance, there are greater associations between maximal speed performance and standing long jump and multiple jump distance.

The result of the meta-analysis indicates that horizontal jump distance positively associates with sprint performance, and the strength of associations ranges from moderate to very large. The positive association demonstrates that an athlete who jumps longer can sprint faster. This result is due to the fact that several similarities are involved in both tasks, such as strength (*e.g.*, the generation of horizontal force and power-output), energy metabolism (*e.g.*, anaerobic energy system), movement characteristics (*e.g.*, unilateral ground contacts and triple joint extension) and so on. Notably, we find that standing long jump shows a greater correlation with the maximal sprint performance than the sprint acceleration performance (*r* = 0.73 VS *r* = 0.45). The study conducted by Robbins & Young (2012) explored positional relationships between sprint and jump ability in elite college football players, the authors reported that correlation coefficients between standing long jump distance and maximum speed performance ranged from 0.35 to 0.47, while its correlation coefficients with acceleration performance were 0.35 to 0.43. Moreover, Mackala, Fostiak & Kowalski (2015) also reported that correlation coefficients between standing long jump and 10 m, 30 m, and 100 m sprint time were 0.70, 0.74 and 0.82, respectively. These findings were in line with our study. Similarly, compared to the sprint acceleration performance, there is a stronger association between multiple jump distance and maximal speed performance (*r* = 0.73 VS *r* = 0.45). However, it is challenging to interpret this result and also it is not the purpose of this review, and we suggest that future studies could explore this issue in depth.

In this review, the highest magnitude of association between horizontal jump and acceleration and maximal speed performance is found in the multiple jump (sprint acceleration: *r* =  − 0.69, maximal speed: *r* =  − 0.76). This finding is in line with a previous study conducted by Chaouachi et al. (2009b), the authors reported that a stronger association was observed between horizontal five jumping and sprint accerelation time (*r* =  − 0.55 ∼  − 0.70) compared to bilateral standing long jump (*r* =  − 0.38 ∼  − 0.45). Notably, six studies included in this review analyzed the associations of the sprint time with multiple jump, only one study assessed double-leg multiple jump test (Mackala et al., 2021), other studies evaluated unilateral multiple-step jumps with foot changes (Chaouachi et al., 2009a; Chaouachi et al., 2009b; Hennessy & Kilty, 2001; Mackala, Fostiak & Kowalski, 2015; Nesser et al., 1996; Washif & Kok, 2020; Yanci et al., 2014), which also called the sprint bounding (a combination of sprinting and bounding) (Young, 1992). The sprint bounding can produce larger horizontal propulsive forces (Mero & Komi, 1994), and emphasize shorter contact time (Mero & Komi, 1994), with a notable reduction of 0.6s in the fifth step compared to the first step (Washif & Kok, 2020), which also occurred in the transition from acceleration to maximal speed. These characteristics of horizontal multiple jump are similar to those of the sprint. However, not all studies are consistent with this finding. For example, Mackala, Fostiak & Kowalski (2015) reported that the sprint acceleration time was correlated greater with standing long jump (*r* =  − 0.70 ∼  − 0.74) than with multiple jump (*r* =  − 0.62 ∼  − 0.71). Moreover, the sprint time of maximum speed was correlated with the standing long jump (*r* =  − 0.82) to a similar extent as it does with the multiple jumps (*r* =  − 0.81 and −0.83). Also, it is difficult for us to explain these conflict results and it is also not the aim of this review, and more studies ought to explore this point.

Regarding the associations between standing triple jump distance and acceleration and maximal sprint performance, it is hard for us to draw an exact conclusion due to the inconsistence results. Of the studies reported relationships between triple jump distance and sprint performance (five studies for acceleration, and two for maximal speed), significant negative associations with the sprint time of acceleration phase and maximal speed phase were reported in 3 and 1 studies, respectively.

Only one study analyzed elite sprinters and assessed the standing triple jump, which was performed unilaterally without a side-change (Habibi et al., 2010). The authors reported that the front-leg and the back-leg standing triple jump were significantly correlated with 10 m sprint time (Habibi et al., 2010). However, the study conducted by Yanci et al. (2014) found that the significant associations between standing triple jump distance and 10 m and 15 m performance only been observed in non-dominant side, not in dominant side in soccer players. Soccer players need to complete intensive actions unilaterally, which resulted in inter-limb asymmetry. Thus, this may partly explain this inconsistent finding. Moreover, we also found inconsistent results on the associations between standing triple jump distance and performance on different sprint distance. For example, within the sprint acceleration phase, non-dominant standing triple jump distance significantly only associated with 10 m and 15 m sprint time, not with 5 m sprint time (Yanci et al., 2014).

Although there is a strong correlation between horizontal jump and sprint performance, there is no direct evidence in this review to support the effectiveness of jump training to improve or assess sprint performance. It is worth noting that there are also significant differences between horizontal jump and sprint. Firstly, athletes need to swing their arms alternately in the sprint, while in the horizontal jumping both arms are usually used to swing at the same time. Moreover, sprinting required a faster speed than horizontal jumping. For example, faster and slower sprinters had blocking speeds of about 3.16 m/s and 3.38 m/s, respectively, with blocking accelerations between 7.35 and 7.47 m/s (Čoh et al., 2017), while standing long jump takeoff speeds only ranged from 1.64 to 1.94 m/s (Wen-Lan et al., 2003). Therefore, although similar characteristics were observed in the horizontal jump and sprint, both sprint and jump are specific tasks and have several distinct determinants that influence their performance.

There are several limitations to this study. First, we did not calculate effect sizes for correlations between horizontal triple-step jump and sprint performance due to high heterogeneity. However, the horizontal triple-step jump test is a traditional tool to assess lower limb horizontal power in a range of sports, including sprint and team sports. Thus, more studies need to analyze this aspect and determine the heterogeneity sources. Secondly, we defined the multiple jump as the number of horizontal jump greater than 3, this classification may influence the accuracy of the correlation results. More work is needed in the future to determine the associations with typical and exact multiple jump (*e.g.*, five and ten horizontal jump).

## Conclusions

This study systematically evaluated the associations between horizontal jump distance and sprint acceleration and maximal speed performance. The sprint time of the sprint acceleration phase showed moderate and negative correlations with standing long jump, horizontal drop and single leg standing long jump distance, and large and negative correlations with multiple jump distance. The sprint time of the maximal speed phase was very largely correlated with standing long jump and multiple jump distance. Moreover, three of five studies assessed the standing triple jump reported significant positive associations with the sprint time of the sprint acceleration phase. Therefore, this review indicates the moderate to very large associations between horizontal jump and sprint acceleration and maximal speed performance, and the highest magnitude of associations between them is found in the multiple jump. Moreover, compared to the sprint acceleration performance, there are greater associations between maximal speed performance and standing long jump and multiple jump distance.

### Studies included in systematic review only

Habibi et al. (2010), Hudgins et al. (2013), Kale et al. (2009), Mackala et al. (2021), Maulder & Cronin (2005), Maulder, Bradshaw & Keogh (2006) and Yanci et al. (2014).

### Studies included in systematic review and meta-analysis

Abbas (2016), Almuzaini & Fleck (2008), Banda et al. (2019), Chaouachi et al. (2009a), Chaouachi et al. (2009b), Dietze-Hermosa et al. (2021), Habibi et al. (2010), Hennessy & Kilty (2001), Holm et al. (2008), Kulakowski et al. (2020), Lockie et al. (2014), Lockie, Dawes & Callaghan (2020), Loturco et al. (2015), Mackala, Fostiak & Kowalski (2015), Mackala et al. (2021), Maulder & Cronin (2005), Maulder, Bradshaw & Keogh (2006), McCurdy et al. (2010), Meylan et al. (2009), Nesser et al. (1996), Popowczak et al. (2019), Robbins & Young (2012), Schuster & Jones (2016), Washif & Kok (2020) and Yanci et al. (2014).

##  Supplemental Information

10.7717/peerj.14650/supp-1 Supplemental Information 1 PRISMA checklist

10.7717/peerj.14650/supp-2 Supplemental Information 2 Funnel plots for bias assessment

10.7717/peerj.14650/supp-3 Supplemental Information 3 Raw data for meta-analysis

10.7717/peerj.14650/supp-4 Supplemental Information 4 The rationale of this review

## Additional Information and Declarations

### Data Availability

The following information was supplied regarding data availability: The raw measurements are available in the Supplementary Files.
