# Supplementary material for: Correlations between horizontal jump and sprint acceleration and maximal speed performance: a systematic review and meta-analysis
Source: PeerJ. 2023 Feb 1;11:e14650. doi: 10.7717/peerj.14650 (PMC9899053; doi:10.7717/peerj.14650)
Supplement: Supplemental Information 1 [file peerj-11-14650-s001.zip › PRISMA DOCUMENTATION/PRISMA_2020_flow_diagram.docx]

**Identification of studies via databases and registers**

Records identified from:

Pubmed (n = 970)

Web of Science (n = 1019)

MEDLINE(EBSCOhost) (n = 826)

References lists (n = 2)

N = 2817

Records removed *before screening*:

Duplicate records removed

(n = 1137)

**Identification**

Potentially relevant papers screened

(n = 1680)

Papers excluded on the basic of titles and abstracts

(n = 1610)

**Screening**

Papers excluded: (n = 42)

-No Pearson’s reported (n= 20)

-No sprint time reported (n = 3)

-No jump distance reported (n = 7)

-Age did not meet the criteria (n =8)

- Non-well-trained subjects (n =4)

Papers assessed for eligibility

(n = 70)

Studies included in review

(n = 28)

**Included**

*Consider, if feasible to do so, reporting the number of records identified from each database or register searched (rather than the total number across all databases/registers).

*From:*  Page MJ, McKenzie JE, Bossuyt PM, Boutron I, Hoffmann TC, Mulrow CD, et al. The PRISMA 2020 statement: an updated guideline for reporting systematic reviews. BMJ 2021;372:n71. doi: 10.1136/bmj.n71

For more information, visit: <http://www.prisma-statement.org/>
