# Supplementary material for: Correlations between horizontal jump and sprint acceleration and maximal speed performance: a systematic review and meta-analysis
Source: PeerJ. 2023 Feb 1;11:e14650. doi: 10.7717/peerj.14650 (PMC9899053; doi:10.7717/peerj.14650)
Supplement: Supplemental Information 2 [file peerj-11-14650-s002.docx]

#
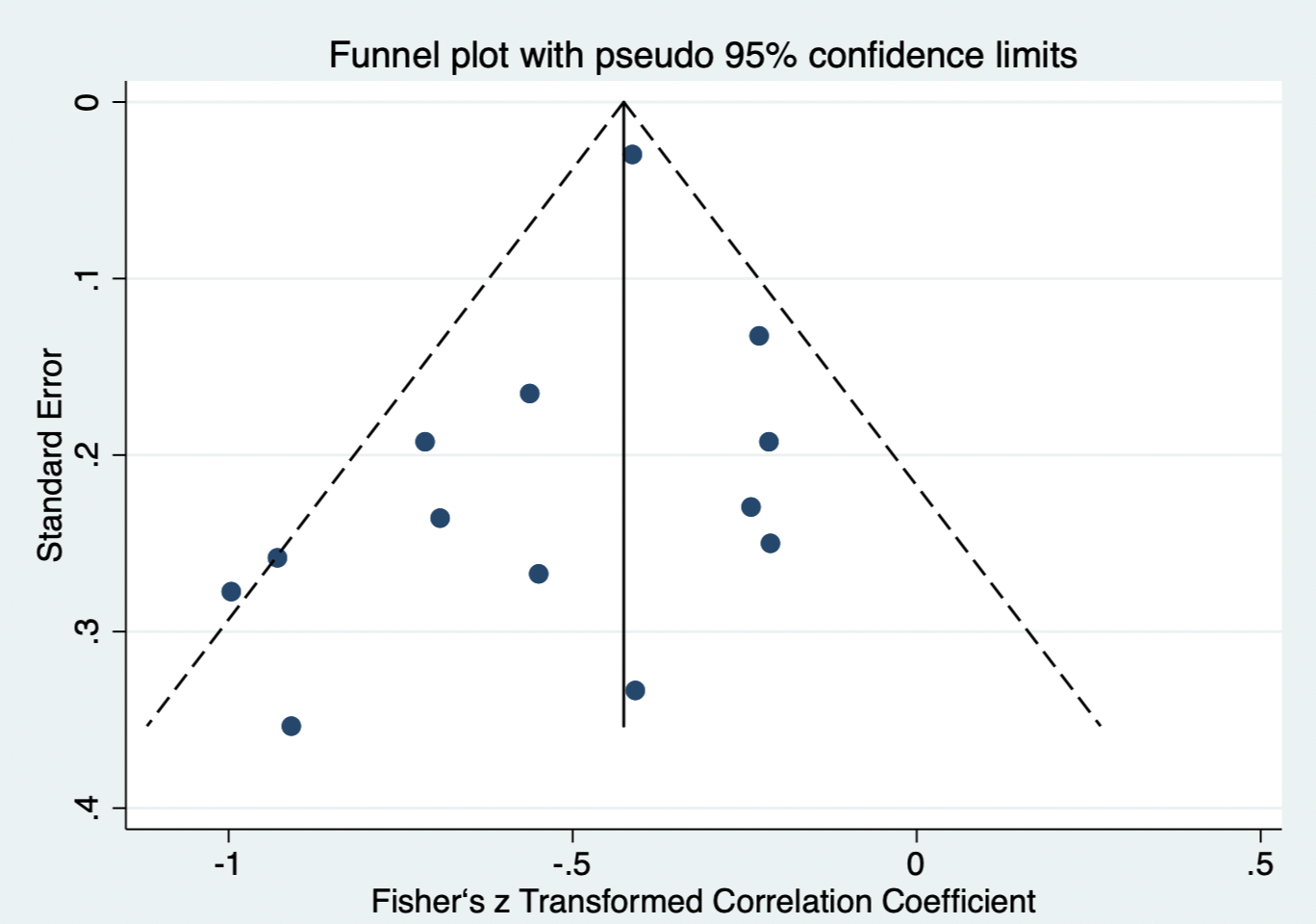
Figure 5. Funnel plot presenting Fisher’s z transformed correlation coefficient between standing long jump and acceleration performance, plotted against its standard error

# Figure 6. Funnel plot presenting Fisher’s z transformed correlation coefficient between standing long jump and maximal speed performance, plotted against its standard error

#
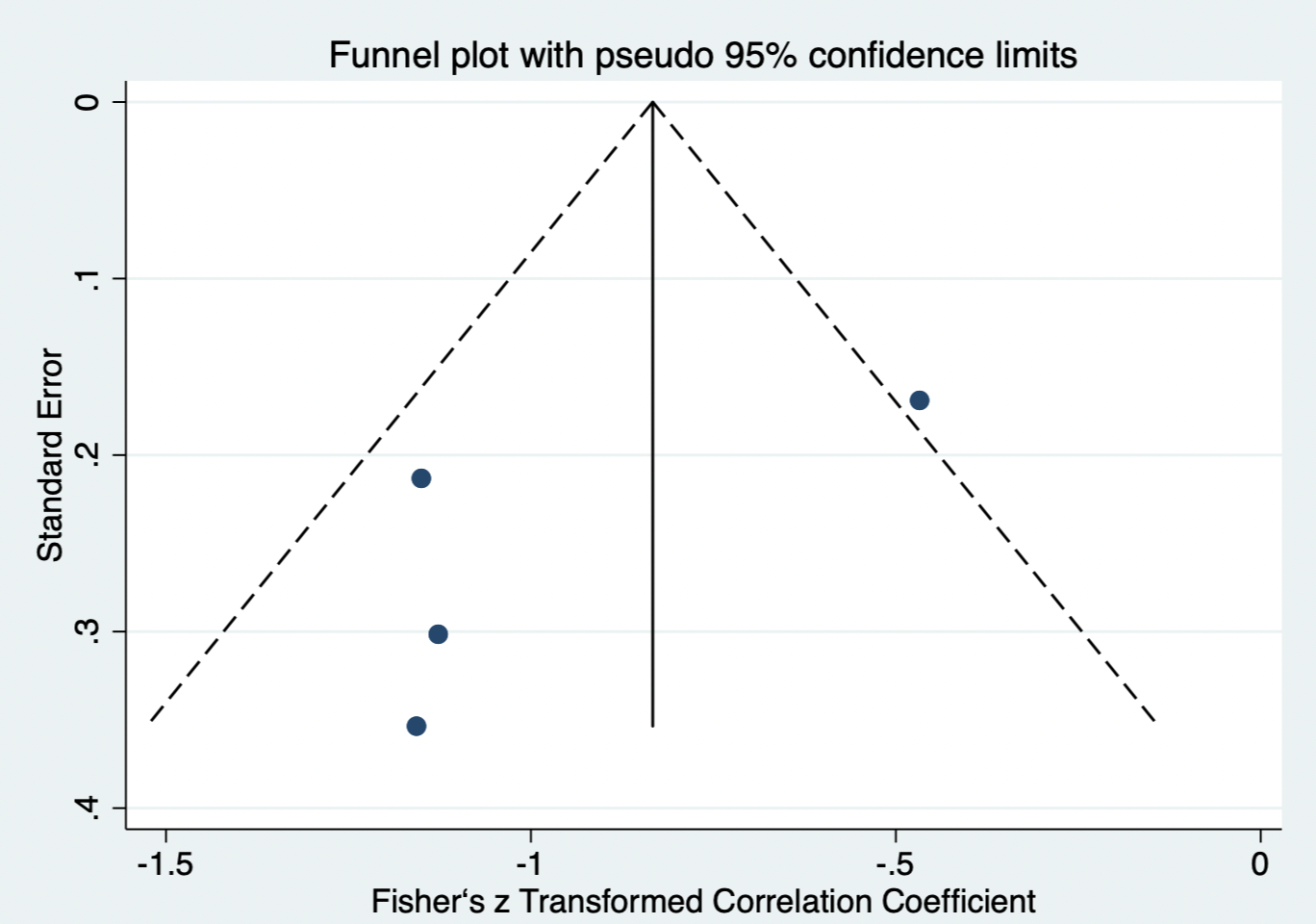


# Figure 7. Funnel plot presenting Fisher’s z transformed correlation coefficient between horizontal drop jump and acceleration performance, plotted against its standard error

#
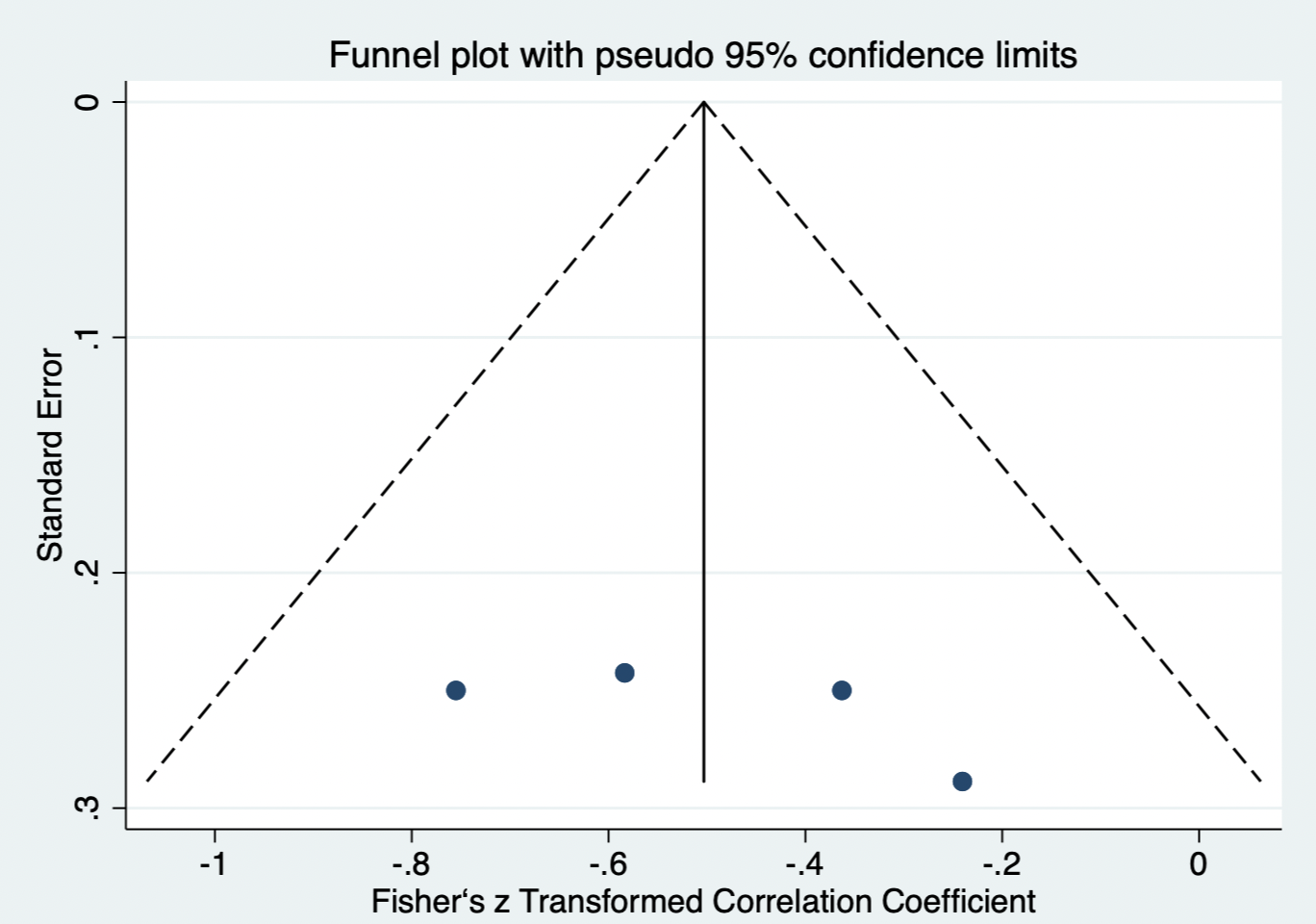


# Figure 8. Funnel plot presenting Fisher’s z transformed correlation coefficient between single-leg standing long jump and acceleration performance, plotted against its standard error


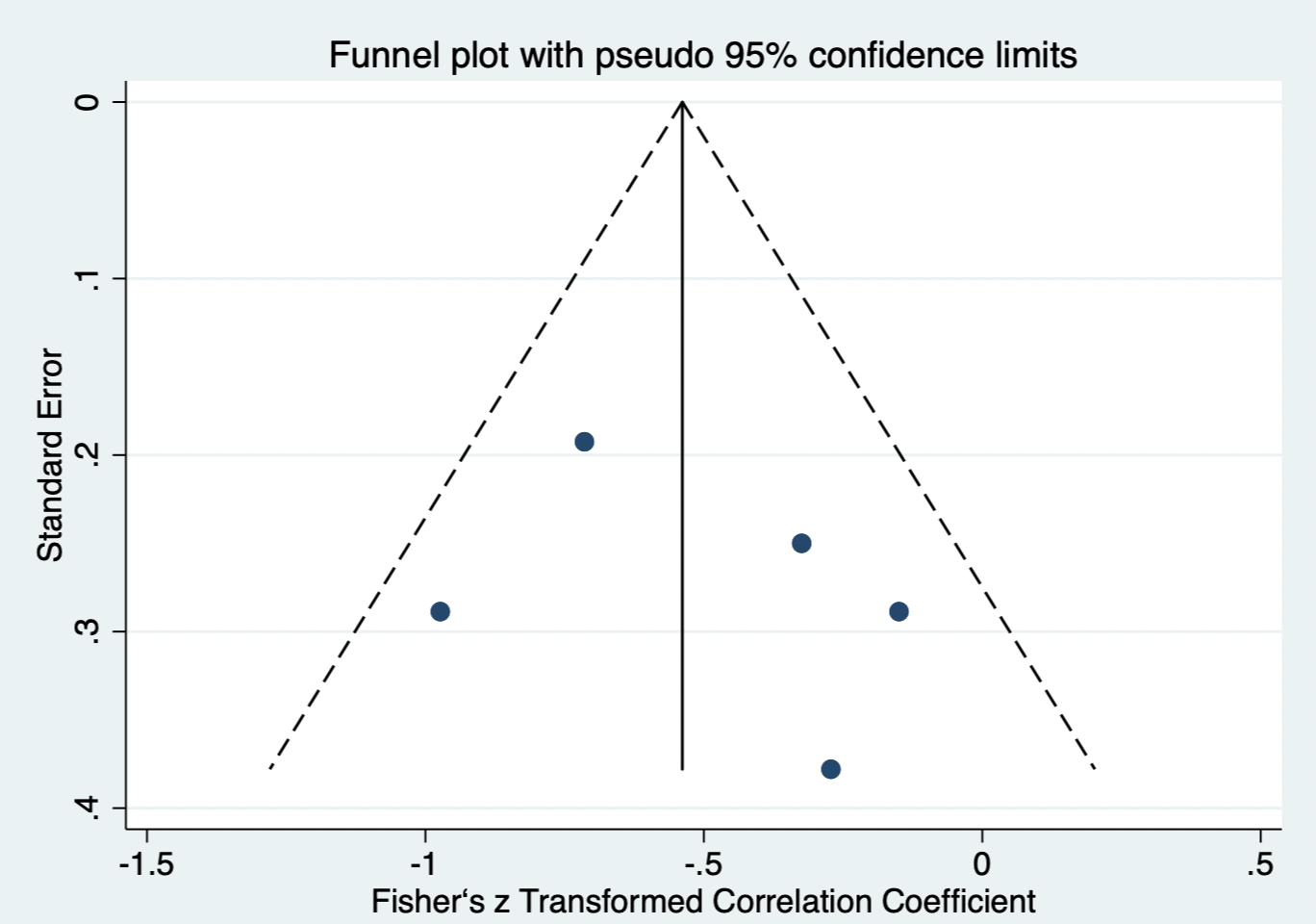


Figure 9. Funnel plot presenting Fisher’s z transformed correlation coefficient between multiple jump and acceleration performance, plotted against its standard error

**
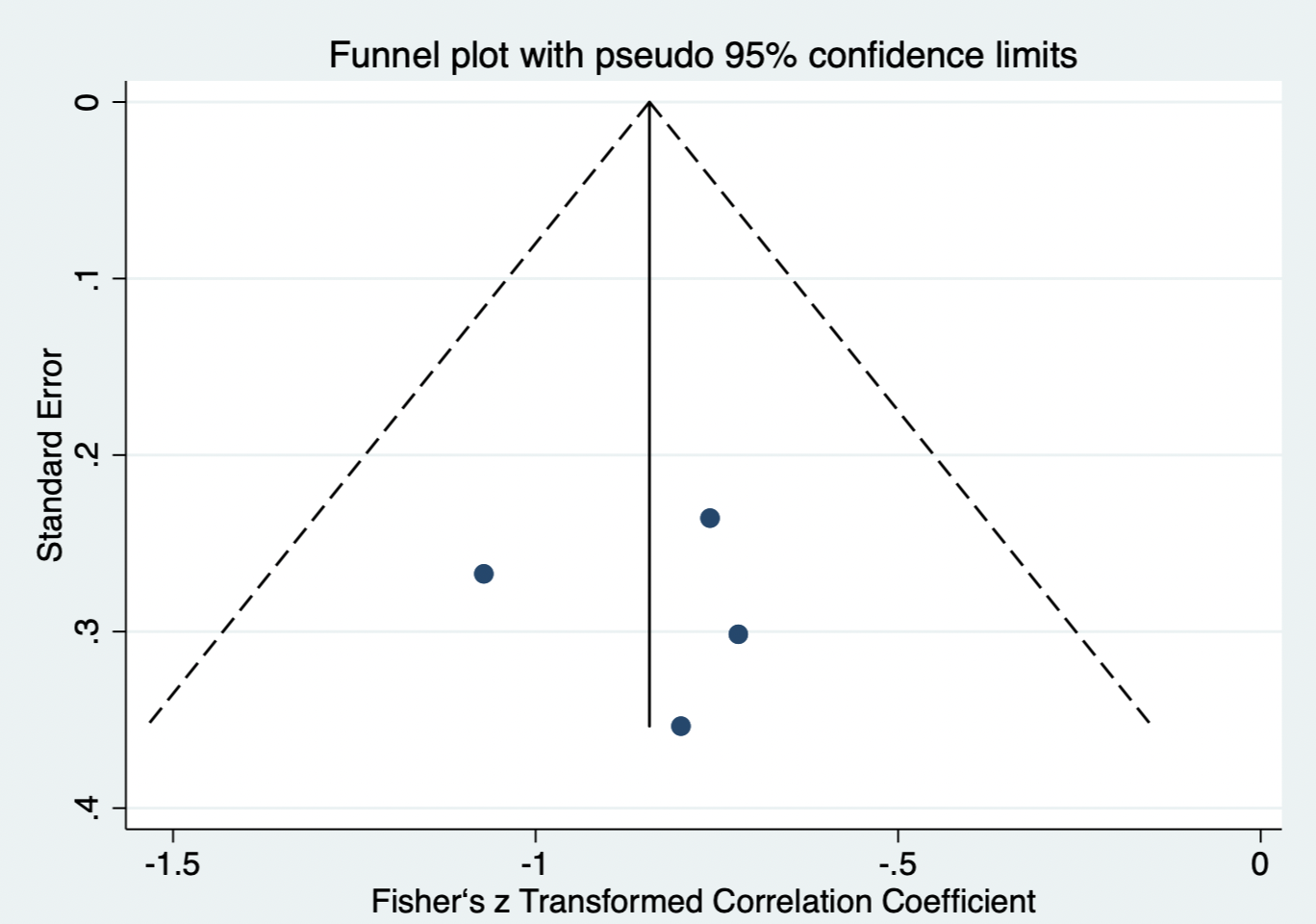
**

Figure 10. Funnel plot presenting Fisher’s z transformed correlation coefficient between multiple jump and maximal speed performance, plotted against its standard error

**
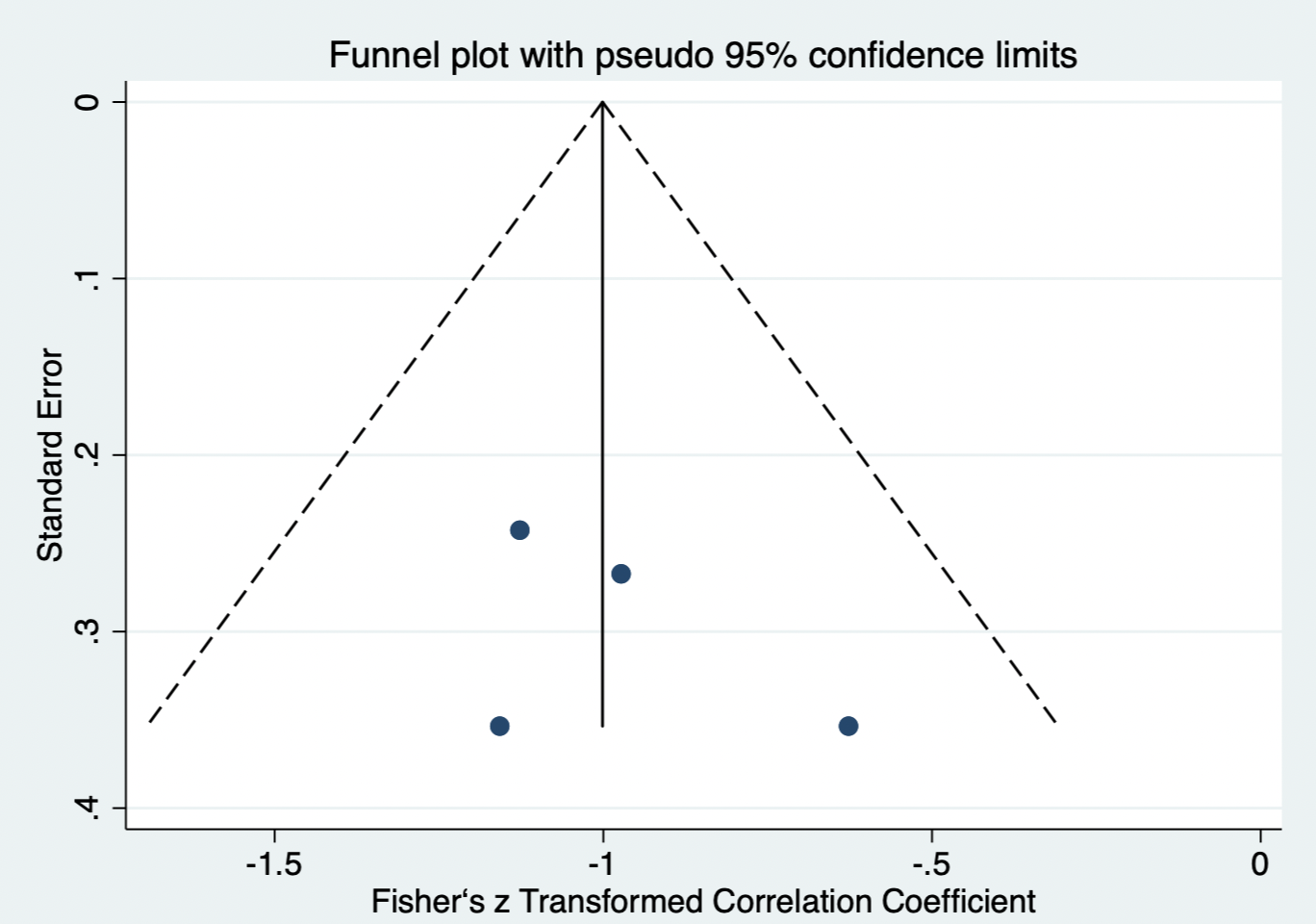
**
