# Supplementary material for: Correlations between horizontal jump and sprint acceleration and maximal speed performance: a systematic review and meta-analysis
Source: PeerJ. 2023 Feb 1;11:e14650. doi: 10.7717/peerj.14650 (PMC9899053; doi:10.7717/peerj.14650)
Supplement: Supplemental Information 4 [file peerj-11-14650-s004.docx]

The rationale for conducting the systematic review / meta-analysis:

Sprint ability is an essential element of athletic performance in a variety of sports. The sprint's most capable abilities were acceleration and maximum speed. Hence, the topic of how to analyze and improve acceleration and maximum speed performance has received a lot of attention. According to the training specificity hypothesis, the evaluation tools and exercises chosen must have some features that are similar to those seen in specific sports. Horizontal jump drills appear to be the optimal strategy for sprinters to evaluate and enhance acceleration and maximal speed performance. The standing long jump, horizontal drop jump, horizontal triple jump, and multi-step jump (steps > 3) are all common horizontal jump drills. However, the abilities reflected in the various horizontal jump tasks varied. Therefore, identifying the correlations between various horizontal jumping drills and sprint performance can help sprinters choose the appropriate tasks to assess and improve their acceleration or maximal speed capability.

The contribution that it makes to knowledge in light of previously published related reports, including other meta-analyses and systematic reviews

To our knowledge, this is the first systematic review to investigate the associations between sprint time and horizontal jump performance. We find that acceleration performance was significantly and moderately correlated with the standing long jump (r=-0.46, z=-7.33, p=0.000), single-leg standing long jump (r=-0.48, z=-3.49, p=0.000) and horizontal drop jump performance (r=-0.46, z=-3.93, p=0.000). Furthermore, there were significant correlations between acceleration and triple jump distance (r=-0.75, z=-6.86, p=0.000). Maximal speed performance was significantly and very largely associated with standing long jump (r=-0.82, z=-7.33, p=0.000) and multiple jump performance (r=-0.73, z=-5.09, p=0.000). Therefore, we suggest that horizontal jump tests are feasible and high effective methods to improve and assess sprint performance.
